# Navigating the new landscape of second‐line treatment in advanced hepatocellular carcinoma

**DOI:** 10.1111/liv.14533

**Published:** 2020-06-10

**Authors:** Lorenza Rimassa, Marcus‐Alexander Wörns

**Affiliations:** ^1^ Medical Oncology and Hematology Unit Humanitas Cancer Center Humanitas Clinical and Research Center – IRCCS Milan Italy; ^2^ Department of Biomedical Sciences Humanitas University Milan Italy; ^3^ Department of Internal Medicine I/Cirrhosis Center Mainz (CCM) University Medical Center of the Johannes Gutenberg‐University Mainz Germany

**Keywords:** cabozantinib, hepatocellular carcinoma, ipilimumab, nivolumab, pembrolizumab, ramucirumab, regorafenib

## Abstract

Sorafenib and lenvatinib are approved for first‐line treatment of patients with advanced hepatocellular carcinoma (HCC), and the efficacy of atezolizumab plus bevacizumab has been demonstrated versus sorafenib. Over time, first‐line treatment frequently fails, and regorafenib, cabozantinib, ramucirumab (for patients with alpha fetoprotein ≥400 ng/mL), nivolumab, pembrolizumab and ipilimumab plus nivolumab are approved for use after sorafenib (but not lenvatinib) treatment in advanced HCC. Given the considerable complexity in the therapeutic landscape, the objective of this review was to summarize the clinical evidence for second‐line agents and provide practical guidance for selecting the best sequential treatment approach. The timing and sequencing of treatment switches are key to optimizing patient outcomes in advanced HCC, and decisions should be informed by reasons for discontinuation of previous therapy and disease progression. It is important not to switch too soon, because sequential treatment benefit may then be lost, nor should switching be delayed too long. Effectiveness, safety and tolerability, patient quality of life, route of administration, dosing regimen, drug class, molecular target and individual patients’ characteristics, including comorbidities, inform the selection of second‐line systemic treatment, independently of the aetiology of HCC, tumour stage and the response to previous treatment. Biomarkers predictive of treatment effectiveness are of great value, but currently biomarker‐driven patient selection is possible only in the case of ramucirumab. The approval of new combination therapies for advanced HCC in the first‐line setting will further increase the complexity of decision‐making. However, the important factors will remain the individual patient’s characteristics and preferences.

**Abbreviations**
AE: adverse event
AFP: alpha fetoprotein
BCLC: Barcelona Clinic Liver Cancer
CI: confidence interval
DCR: disease control rate
EASL: European Association for the Study of the Liver
ECOG: Eastern Cooperative Oncology Group
ESMO: European Society for Medical Oncology
FACT: Functional Assessment of Cancer Therapy
FDA: Food and Drug Administration
HCC: hepatocellular carcinoma
HR: hazard ratio
HRQoL: health‐related quality of life
ORR: objective response rate
OS: overall survival
PD‐1: programmed death receptor‐1
PD‐L1: programmed death‐ligand 1
PFS: progression‐free survival
TTP: time to progression
VEGF: vascular endothelial growth factor

**Key points**

The number of treatment options for patients with advanced hepatocellular carcinoma has increased in recent years and is expected to grow further. The increase in treatment options has created complexity in the therapeutic landscape, especially in terms of second‐line treatment options and timing/sequencing of switches. This review provides a summary of the clinical evidence for approved second‐line agents (regorafenib, cabozantinib, ramucirumab, nivolumab [including in combination with ipilimumab] and pembrolizumab) and guidance for selecting the best treatment strategy. Factors informing treatment decisions for advanced hepatocellular carcinoma, and open issues about biomarkers and the expected approval of new combination therapies, are discussed.

## 1 INTRODUCTION

Liver cancer is the fifth most common cancer and the second most frequent cause of cancer‐related mortality worldwide.^1^ Hepatocellular carcinoma (HCC) accounts for 90% of primary liver cancer and occurs predominantly in patients with underlying chronic liver disease and cirrhosis.^2^ The treatment of HCC depends on the stage of the disease, usually based on the Barcelona Clinic Liver Cancer (BCLC) model, which considers factors that impact prognosis, such as tumour burden, liver function and performance status.^2^, ^3^ In patients with unresectable intermediate (BCLC stage B) or advanced (BCLC stage C) HCC, noncurative interventions, which attempt to prolong survival by slowing tumour progression, are used. These include transarterial chemoembolization for patients with intermediate HCC, and systemic therapy with targeted or immunotherapeutic agents for patients with intermediate HCC (through treatment stage migration; unsuitable for locoregional therapy) or advanced HCC.^4^

In 2007, sorafenib, a multi‐kinase inhibitor, became the first systemic therapy approved for first‐line treatment of unresectable HCC in the EU and in the USA.^5^, ^6^ Approval of sorafenib was based on efficacy data from two randomized, double‐blind, placebo‐controlled, phase 3 trials in which sorafenib significantly prolonged overall survival (OS) vs placebo in patients with advanced HCC who had not received prior systemic treatment.^7^, ^8^ In 2018, the tyrosine kinase inhibitor lenvatinib was approved as an alternative to sorafenib for first‐line treatment of advanced or unresectable HCC,^9^, ^10^ based on phase 3 trial data showing non‐inferiority to sorafenib in the first‐line setting.^11^ In the 2018 European Association for the Study of the Liver (EASL) and European Society for Medical Oncology (ESMO) treatment guidelines (published since the approval of lenvatinib), both sorafenib and lenvatinib are recommended as first‐line treatment options in patients with advanced HCC.^2^, ^12^, ^13^ More recently, results of the phase 3 trial evaluating atezolizumab in combination with bevacizumab in the treatment of patients with unresectable HCC, who had not received prior systemic therapy, were presented in November 2019. Both of the study’s co‐primary endpoints were met, demonstrating improvements in OS and progression‐free survival (PFS) compared with sorafenib.^14^

First‐line treatment in advanced HCC frequently fails after a period of time, owing to adaptive or intrinsic resistance, disease progression or significant toxicity, and thus there is a need for second‐ and later‐line treatment options. Several potential second‐line treatments, including tivantinib, brivanib and everolimus, have failed to show a survival benefit over placebo in phase 3 clinical trials (Supplemental Table S1).^15^, ^16^, ^17^, ^18^, ^19^, ^20^, ^23^ Regorafenib, a multi‐kinase inhibitor, was the first agent to show a survival benefit over placebo in patients progressing on sorafenib and was approved for the treatment of advanced HCC after prior sorafenib treatment in the USA in April 2017^24^ and in the EU in August 2017.^25^ Approval for use after prior sorafenib treatment followed for cabozantinib (in the EU in November 2018^26^ and in the USA in January 2019^27^), an inhibitor of vascular endothelial growth factor (VEGF) receptors and the MET and TAM kinases, and, for patients with alpha fetoprotein (AFP) ≥400 ng/mL, for ramucirumab (in the USA in May 2019^28^ and in the EU in August 2019^29^), an antiangiogenic monoclonal antibody that targets the VEGF receptor‐2. Two immunotherapies targeting programmed death receptor‐1 (PD‐1) received accelerated approvals as second‐line therapies in the USA on the basis of their phase 1/2 trial results: nivolumab in September 2017^30^ and pembrolizumab in November 2018.^31^ In subsequent phase 3 trials of nivolumab in first‐line^32^ and pembrolizumab in second‐line^20^ settings, these therapies did not meet their primary endpoints. Any impact of these trial results on approval status by the Food and Drug Administration (FDA) is awaited. In March 2020, ipilimumab, a human cytotoxic T‐lymphocyte antigen 4‐blocking antibody, was approved in the USA, in combination with nivolumab, for patients with HCC who have been previously treated with sorafenib, based on data from a phase 1/2 trial.^33^

This rapid proliferation of second‐line treatment options brings new hope for the treatment of advanced HCC. However, the approval of these new therapies creates complexity in the therapeutic landscape for treating clinicians, who need to understand the different risks and benefits associated with various systemic therapies so that they can choose the most appropriate treatment option for their patients. For example, regorafenib, cabozantinib and ramucirumab are indicated in advanced HCC for patients previously treated with sorafenib but not for patients previously treated with lenvatinib, for whom sorafenib is sometimes used as a subsequent treatment option.^34^ An objective of this review was to describe factors for consideration by clinicians when assessing the evidence for the available second‐line systemic agents and selecting the best sequential treatment strategy for their patients with advanced HCC.

## 2 SUMMARY OF DATA FOR APPROVED SECOND‐LINE SYSTEMIC AGENTS

To identify relevant evidence on second‐line treatment options after sorafenib and lenvatinib in advanced HCC from phase 2 and phase 3 clinical trials, structured searches of the published literature were conducted in August 2019. Potentially relevant English‐language articles in peer‐reviewed journals were identified by using the PubMed interface to search MEDLINE and related biomedical content with terms listed in Supplemental Table S2. Potentially relevant congress abstracts published during the previous 5 years were identified by searching Embase using equivalent terms, and additional pragmatic searches for recently published evidence were conducted through to April 2020. Articles identified by the searches were screened manually to evaluate their relevance for inclusion in this review.

An overview of product characteristics is provided in Table 1. Pivotal clinical trial data are provided in Tables 2, ^37^, ^49^, ^61^, ^69^, ^71^, ^72^ and 3,^37^, ^49^, ^59^, ^61^, ^69^, ^71^, ^72^ covering trial design and efficacy outcomes, and safety/tolerability and health‐related quality of life (HRQoL) respectively.

**Table 1:** Characteristics of agents approved for second‐line treatment of patients with advanced HCC

	Regorafenib	Cabozantinib	Ramucirumab	Nivolumab	Pembrolizumab	Ipilimumab plus nivolumab
Drug class	Multitarget kinase inhibitor	Multitarget kinase inhibitor	Monoclonal antibody	Monoclonal antibody	Monoclonal antibody	Monoclonal antibodies
Molecular targets	VEGFR‐1‐3, TIE2, KIT, RET, RAF1, BRAF, BRAFV600E, PDGFR, FGFR	VEGFR‐2, MET, RET, AXL, FLT3, c‐KIT	VEGFR‐2	PD‐1	PD‐1	Nivolumab: PD‐1 Ipilimumab: CTLA‐4
Route of administration	Oral, with food	Oral, not with food (administer ≥2 h after and ≥1 h before eating)	Intravenous infusion	Intravenous infusion	Intravenous infusion	Intravenous infusion
Dosing schedule	160 mg once daily for the first 21 d of 28‐day cycle	60 mg once daily	8 mg/kg every 2 wk	240 mg every 2 wk or 480 mg every 4 wk	200 mg every 3 wk	Nivolumab 1 mg/kg followed by ipilimumab 3 mg/kg on the same day every 3 wk for 4 doses, then nivolumab 240 mg every 2 wk or 480 mg every 4 wk
Approved indication (2L HCC) in EU/USA	Patients previously treated with sorafenib	Patients previously treated with sorafenib	Patients who have AFP ≥400 ng/mL and have been previously treated with sorafenib	Patients previously treated with sorafenib (not approved for HCC in EU)	Patients previously treated with sorafenib (not approved for HCC in EU)	Patients previously treated with sorafenib (not approved for HCC in EU)

Sources: Stivarga EU SmPC^25^; Stivarga USPI^24^; Cabometyx EU SmPC^26^; Cabometyx USPI^27^; Opdivo USPI^30^; Keytruda USPI^31^; Cyramza USPI^28^; Yervoy USPI.^33^

Abbreviations: 2L, second‐line; AFP, alpha fetoprotein; CTLA‐4, cytotoxic T‐lymphocyte antigen 4; FGFR, fibroblast growth factor receptor; HCC, hepatocellular carcinoma; PD1, programmed death receptor‐1; PDGFR, platelet‐derived growth factor receptor; SmPC, summary of product characteristics; USPI, United States prescribing information; VEGFR, vascular endothelial growth factor receptor.

**Table 2:** Pivotal clinical trials for agents approved for second‐line treatment of patients with advanced HCC: study design and efficacy data

	Regorafenib	Cabozantinib	Ramucirumab	Nivolumab	Pembrolizumab	Ipilimumab plus nivolumab^a^
Study	RESORCE (NCT01774344) Bruix *et al*. 2017^37^	CELESTIAL (NCT01908426) Abou‐Alfa *et al*. 2018^49^	REACH‐2 (NCT02435433) Zhu *et al*. 2019^61^	CheckMate 040 (NCT01658878) El‐Khoueiry *et al*. 2017^69^	KEYNOTE‐224 (NCT02702414) Zhu *et al*. 2018^72^	CheckMate 040 (NCT01658878) Yau *et al*. 2019^71^
Design	Phase 3 double‐blind RCT vs placebo	Phase 3 double‐blind RCT vs placebo	Phase 3 double‐blind RCT vs placebo	Phase 1‐2 open‐label, non‐comparative, dose‐escalation and expansion trial	Phase 2 open‐label, non‐comparative trial	Phase 1‐2 open‐label, non‐comparative trial
Primary endpoint	OS	OS	OS	Dose‐escalation phase: safety and tolerability Dose‐ expansion phase: ORR	ORR	Safety and tolerability and ORR
Patients (N)	573	707	292 with elevated baseline AFP	262 (48 for dose escalation, 214 for dose‐ expansion)	104	50
Previous treatment	Sorafenib (2L)	One or two prior systemic treatments for HCC, including sorafenib (2L or 3L)	Sorafenib (2L)	Previous sorafenib allowed (1L and 2L)	Sorafenib (2L)	Previous sorafenib allowed (1L and 2L)
CP class	A	A	A	A (or B7 for dose escalation)	A	A
ECOG PS	0 or 1	0 or 1	0 or 1	0 or 1	0 or 1	0 or 1
Efficacy						
Median OS, months	10.6 vs 7.8, HR 0.63 (95% CI 0.50‐0.79), *P* < 0.0001	10.2 vs 8.0, HR 0.76 (95% CI 0.63‐0.92), *P* = 0.005 (2L subgroup, 11.3 vs 7.2, HR 0.70 [95% CI 0.55‐0.88])	8.5 vs 7.3, HR 0.71 (95% CI 0.53‐0.95), *P* = 0.0199	6‐ and 9‐month rates in dose expansion: 83% and 74%, respectively	12.9 (95% CI 9.7‐15.5)	23 (95% CI 9‐NA)
Median PFS, months	3.1 vs 1.5, HR 0.46 (95% CI 0.37‐0.56), *P* < 0.0001	5.2 vs 1.9, HR 0.44 (95% CI 0.36‐0.52), *P* < 0.001 (2L subgroup, 5.5 vs 1.9, HR 0.40 [95% CI 0.32‐0.50])	2.8 vs 1.6, HR 0.45, (95% CI 0.34‐0.60), *P* < 0.0001	4.0 in dose expansion	4.9 (95% CI 3.4‐7.2)	NR
Median TTP, months	3.2 vs 1.5, HR 0.44 (95% CI 0.36‐0.55) *P* < 0.0001	5.4 vs 1.9, HR 0.41 (95% CI 0.34‐0.49),	3.0 vs 1.6, HR 0.43 (95% CI 0.31‐0.58), *P* < 0.0001	4.1 in dose expansion	4.9 (95% CI 3.9‐8.0)	NR
ORR, %	10.6 vs 4.1, *P* = 0.0047 (modified RECIST)	4.0 vs 0.4, *P* = 0.0086 (RECIST v1.1)	4.6 vs 1, *P* = 0.1697 (RECIST v1.1)	20 in dose expansion (RECIST v1.1)	17 (95% CI 11‐26) (RECIST v1.1)	32 (BICR per RECIST v1.1)
DCR, %	65.2 vs 36.1, *P* < 0.001	64 vs 33	59.9 vs 38.9, *P* = 0.0006	64 in dose expansion	62 (95% CI 52‐71)	54 (95% CI 39‐68)

Abbreviations: 1L, first‐line; 2L, second‐line; 3L, third‐line; AFP, alpha fetoprotein; BICR, blinded independent central review; CI, confidence interval; CP, Child‐Pugh; DCR, disease control rate; ECOG PS, Eastern Cooperative Oncology Group Performance Status; FDA, Food and Drug Administration; HCC, hepatocellular carcinoma; HR, hazard ratio; NA, not available; ORR, objective response rate; OS, overall survival; PFS, progression‐free survival; RCT, randomized controlled trial; RECIST, Response Evaluation Criteria In Solid Tumours; TTP, time to progression.

^a^ Results provided are for study Arm A, in which patients received the dosing regimen subsequently approved by the FDA.

**Table 3:** Pivotal clinical trials for agents approved for second‐line treatment of patients with advanced HCC: safety and tolerability and HRQoL data

	Regorafenib	Cabozantinib	Ramucirumab	Nivolumab	Pembrolizumab	Ipilimumab plus nivolumab^a^
Study	RESORCE (NCT01774344) Bruix *et al*. 2017^37^	CELESTIAL (NCT01908426) Abou‐Alfa *et al*. 2018^49^	REACH‐2 (NCT02435433) Zhu *et al*. 2019^61^	CheckMate 040 (NCT01658878) El‐Khoueiry *et al*. 2017^69^	KEYNOTE‐224 (NCT02702414) Zhu *et al*. 2018^72^	CheckMate 040 (NCT01658878) Yau *et al*. 2019^71^
Patients (N)	573	707	292 with elevated baseline AFP	262 (48 in dose escalation, 214 in dose expansion)	104	146
Safety and tolerability						
Patients with ≥1 AEs, %	100 vs 93	99 vs 92	97.0 vs 86.3	83 (treatment‐related in dose escalation)	97	82 (treatment‐related)
Patients with ≥1 SAEs, %	44 vs 47	68 vs 36	58.9 vs 44.2	25 (treatment‐related in dose escalation)	40	37 (treatment‐related)
Patients who discontinued for treatment‐related AEs, %	10 vs 4	16 vs 3	10.7 vs 3.2	3 (across both phases)	5	5
Any AE‐related dose modification, %	68 vs 31	62 vs 13	34.5 vs 13.7	NR	17	NR
Median duration of treatment	3.6 months vs 1.9 months	3.8 months vs 2.0 months	6 vs 4 cycles	NR	4.2 months	NR
HRQoL	No clinically meaningful difference	Clinically and statistically significant benefit in mean QALYs^49^ and significantly more time without disease symptoms and toxicity^59^	No difference in median time to deterioration in FHSI‐8 total score and ECOG PS	No significant change from baseline	NR	NR

Abbreviations: AE, adverse event; AFP, alpha fetoprotein; ECOG PS, Eastern Cooperative Oncology Group Performance Status; FHSI‐8, Functional Assessment of Cancer Therapy Hepatobiliary Symptom Index 8; HCC, hepatocellular carcinoma; HRQoL, health‐related quality of life; NR, not reported/not reached; QALY, quality‐adjusted life‐year; SAE, serious adverse event.

^a^ Results provided are for all patients who received ipilimumab plus nivolumab, with any of the three dosing regimens used in CheckMate 040.

### 2.1 Regorafenib

Regorafenib is an orally administered multi‐kinase inhibitor that is structurally very similar to sorafenib but with a distinct target profile, including additional inhibition of fibroblast growth factor receptor kinases.^35^, ^36^ It was approved for the treatment of patients with HCC who have been previously treated with sorafenib on the basis of the results of the phase 3 RESORCE trial.^37^ RESORCE was a randomized, double‐blind, parallel‐group trial that included 573 adult patients with advanced HCC and Child‐Pugh liver function class A who tolerated sorafenib (≥400 mg/d for ≥20 days of the last 28 days of treatment) and who had documented radiological progression during sorafenib treatment. Patients were randomized 2:1 to regorafenib or placebo within 10 weeks of their last dose of sorafenib, with stratification by geographical region (Asia vs rest of the world), macrovascular invasion (yes vs no) and extrahepatic disease (yes vs no), AFP concentration (<400 vs ≥400 ng/mL) and Eastern Cooperative Oncology Group (ECOG) Performance Status (0 vs 1).

The RESORCE trial included only patients who tolerated sorafenib, with the median duration of prior treatment with sorafenib being 7.8 months.^37^ In these patients, regorafenib significantly increased median OS vs placebo (10.6 vs 7.8 months; hazard ratio [HR] 0.63, 95% confidence interval [CI] 0.50‐0.79; *P*<0.0001). Regorafenib also demonstrated statistically significant superiority to placebo in PFS, time to progression (TTP), objective response rate (ORR) and disease control rate (DCR) (Table 2). Improvement in OS with regorafenib vs placebo was maintained across all pre‐planned subgroup analyses, and there was also consistent benefit in PFS and TTP. Regorafenib has also been found to be efficacious regardless of the pattern of progression on prior sorafenib^38^ or the last sorafenib dose.^39^ An exploratory analysis showed that OS from the start of sorafenib therapy was 26.0 months in patients switched to regorafenib and 19.2 months in patients switched to placebo.^39^ The safety of regorafenib in HCC was found to be consistent with its safety profile in other gastrointestinal malignancies, with no new safety concerns identified, including in subgroup analyses of Chinese patients,^40^ and of patients with low, intermediate or high exposure to regorafenib.^41^ HRQoL was assessed as a tertiary outcome using the Functional Assessment of Cancer Therapy (FACT) General and Hepatobiliary questionnaires and the European Quality of Life 5‐dimension and visual analogue scales, with no clinically meaningful differences found between patients treated with regorafenib and patients receiving placebo. Patients with high levels of AFP, who generally have a poor prognosis, may also benefit from regorafenib treatment, and exploratory analysis of data from RESORCE found an AFP response was associated with improved OS (13.8 months with response vs 9.8 months without response [regorafenib and placebo arms combined]; HR 0.57, 95% CI 0.40‐0.82).^42^ As is the case with sorafenib, better outcomes with regorafenib have been shown to be associated with early onset of dermatological toxicity. A *post hoc* analysis of data from RESORCE found that patients who had a hand‐foot skin reaction event during the first cycle of regorafenib treatment also had improved median OS vs those who did not have such an event (13.2 vs 8.5 months; HR 0.66, 95% CI 0.51‐0.86).^43^

The 2018 EASL guidelines for the management of HCC recommend regorafenib for second‐line treatment of patients with advanced HCC.^2^ The current 2018 ESMO Clinical Practice Guidelines for diagnosis, treatment and follow‐up of HCC identify regorafenib as the standard of care for patients with advanced HCC who have tolerated sorafenib but progressed; it is recommended in patients with well‐preserved liver function and ECOG Performance Status 0 or 1.^12^ Regorafenib is a category 1 option for patients with Child‐Pugh liver function class A who have disease progression on or after sorafenib in the guidelines of the USA’s National Comprehensive Cancer Network (NCCN).^44^

### 2.2 Cabozantinib

Cabozantinib is an orally administered tyrosine kinase inhibitor with activity against a broad range of targets, including VEGF receptor‐2, MET, RET, AXL, FLT3 and c‐KIT.^45^, ^46^ Elevated MET expression is associated with sorafenib treatment and resistance, making it a promising target for second‐line treatment of patients with HCC.^47^, ^48^ Cabozantinib was approved for the treatment of patients with advanced HCC who have been previously treated with sorafenib on the basis of the results of the phase 3 CELESTIAL trial.^49^, ^50^ CELESTIAL was a randomized, double‐blind trial that included 707 adult patients with advanced HCC and Child‐Pugh liver function class A. The CELESTIAL trial included patients who showed radiological progression or were intolerant to prior sorafenib treatment. In addition, patients could have received up to two previous systemic treatments. Overall, 72% of patients included in CELESTIAL were second line and had received only prior sorafenib treatment. Patients were randomized 2:1 to cabozantinib or placebo, with stratification by geographical region (Asia vs rest of the world), evidence of extrahepatic spread of disease or macrovascular invasion or both (yes vs no), and aetiological factors (hepatitis B virus with or without hepatitis C virus; hepatitis C virus without hepatitis B virus; or other).

In CELESTIAL, cabozantinib significantly increased median OS vs placebo (10.2 vs 8.0 months; HR 0.76, 95% CI 0.63‐0.92; *P*=0.005). In the subgroup of patients whose only previous systemic therapy was sorafenib, median OS was 11.3 months with cabozantinib and 7.2 months with placebo (HR 0.70, 95% CI 0.55‐0.88). Cabozantinib also demonstrated statistically significant superiority to placebo in PFS and ORR (Table 2). Cabozantinib may be considered an ‘all‐comer’ second‐line treatment for patients with advanced HCC because benefit vs placebo was shown in patients who showed radiological progression or were intolerant to prior sorafenib treatment consistently across multiple subgroup analyses, including those of aetiological factors, demographic characteristics and treatment history.^49^, ^51^, ^52^, ^53^, ^54^, ^55^ This is in contrast to regorafenib (benefit demonstrated in patients who showed radiological progression only [see above]) and ramucirumab (benefit demonstrated in patients with elevated AFP only [see below]). Patients with high levels of AFP may also benefit from cabozantinib treatment, and in CELESTIAL AFP response was associated with longer OS and PFS with cabozantinib.^56^ A retrospective multivariate analysis of data from CELESTIAL, which included baseline prognostic factors, also found that the development of either grade ≥3 hypertension or any grade hand‐foot skin reaction was associated with prolonged OS and PFS.^57^ The safety and tolerability profile of cabozantinib in patients with advanced HCC is generally in line with those of other tyrosine kinase inhibitors. However, based on naïve comparison, discontinuations due to treatment‐related adverse events (AEs) were higher with cabozantinib in CELESTIAL (16%) than with regorafenib in RESORCE (10%) and ramucirumab in REACH‐2 (10.7%). The inclusion of patients receiving third‐line treatment in CELESTIAL may have contributed to this difference in treatment‐related AEs, as may the inclusion, in contrast to RESORCE, of patients who had not tolerated sorafenib. Cabozantinib was associated with a clinically and statistically significant benefit in mean quality‐adjusted life‐years vs placebo, resulting in part from improved OS and despite an initial, small reduction in health utility. With continued cabozantinib treatment, health utility increased.^58^ A retrospective analysis of data from CELESTIAL found that patients receiving cabozantinib after sorafenib spent significantly more time without disease symptoms and toxicity than those receiving placebo, despite an increase in days with grade 3/4 toxicity before progression.^59^

The 2018 EASL guidelines for the management of HCC recommend cabozantinib for second‐line treatment of patients with advanced HCC.^2^ In addition, the current 2018 ESMO Clinical Practice Guidelines recommend that cabozantinib can be considered for patients with well‐preserved liver function and ECOG Performance Status 0 or 1 who had progressive disease on one or two systemic therapies.^12^ Cabozantinib is a category 1 option for patients with Child‐Pugh liver function class A who have disease progression on or after sorafenib in the guidelines of the NCCN.^44^

### 2.3 Ramucirumab

Ramucirumab is an immunoglobulin G1 monoclonal antibody that targets the VEGF receptor‐2 and is administered by intravenous infusion. Following a previous trial (REACH), in which benefit in comparison with placebo was only seen in a subgroup of patients with AFP ≥400 ng/mL,^23^, ^60^ data from the double‐blind phase 3 trial REACH‐2, including only patients with AFP ≥400 ng/mL at baseline (N=292, randomized 2:1 to ramucirumab or placebo), provided the basis for the European Medicines Agency and FDA approval of ramucirumab to treat this subgroup of patients with advanced HCC who switched from sorafenib.^61^ Randomization was stratified by geographical region (America, Europe, Australia, Israel vs Asia [excluding Japan] vs Japan), macrovascular invasion (yes vs no) and ECOG Performance Status (0 vs 1). In REACH‐2, ramucirumab significantly increased OS vs placebo (8.5 vs 7.3 months; HR 0.71, 95% CI 0.531‐0.949; *P*=0.0199). Ramucirumab also demonstrated statistically significant superiority to placebo in PFS, TTP and DCR (Table 2). All subgroup analyses (based on sex, age [<65 years vs ≥65 years], race, geographical region, aetiology of liver disease, extrahepatic metastases, macrovascular invasion, BCLC score, ECOG Performance Status, previous locoregional therapy and reason for discontinuation of sorafenib) of OS and PFS favoured treatment with ramucirumab. The changes in AFP levels were associated with TTP and OS, and ramucirumab was found to increase time to AFP progression and radiographic TTP and to slow the rate of AFP increase during treatment.^62^ Subgroup analyses of data pooled from REACH and REACH‐2 have further confirmed the treatment benefit of ramucirumab in patients with advanced HCC who switched from sorafenib with elevated AFP,^63^ including specifically in patients in Japan,^64^ and regardless of aetiology of liver disease.^65^ A *post hoc* analysis of data from REACH, REACH‐2 and the pooled population found that treatment with ramucirumab increased OS vs placebo in all radiological progression pattern subgroups.^66^ Median time to deterioration in FACT Hepatobiliary Symptom Index 8 total score and ECOG Performance Status did not differ between patients receiving ramucirumab and those receiving placebo in REACH‐2.^61^ However, analyses of individual patient data pooled from REACH‐2 and patients with AFP ≥400 ng/mL who were included in REACH demonstrated a benefit of ramucirumab in delaying symptom deterioration.^61^, ^64^

The 2018 ESMO Clinical Practice Guidelines for diagnosis, treatment and follow‐up of HCC recommend that ramucirumab can be considered as second‐line treatment after sorafenib of patients with baseline AFP ≥400 ng/mL, well‐preserved liver function and ECOG Performance Status 0 or 1.^12^ Ramucirumab is recommended by the NCCN panel as a category 1 option for patients with a baseline AFP level of ≥400 ng/mL who have disease progression on or after systemic sorafenib treatment.^44^

### 2.4 Nivolumab, ipilimumab plus nivolumab and pembrolizumab

Nivolumab is a recombinant anti‐PD‐1 monoclonal antibody that is administered by intravenous infusion. It was approved by the FDA for the treatment of patients with HCC who have been previously treated with sorafenib based on the results of the single‐arm phase 1/2 CheckMate 040 trial, which included both a dose‐escalation phase and a dose‐expansion phase.^30^, ^67^, ^68^, ^69^ In CheckMate 040, the ORR and DCR with nivolumab were 20% and 64% respectively. Encouraging survival data (Table 2) have continued to be seen through 18 months of treatment.^70^ However, nivolumab has also been investigated as a potential first‐line systemic treatment for patients with HCC in the phase 3 CheckMate 459 trial, and this trial did not meet its primary endpoint: OS was not statistically significantly superior in patients treated with nivolumab vs those treated with sorafenib (HR 0.85, 95% CI 0.72‐1.02; *P*=0.0752).^32^

Ipilimumab is a monoclonal antibody that targets human cytotoxic T‐lymphocyte antigen 4, and which is administered by intravenous infusion. It was recently approved by the FDA, in combination with nivolumab, for the treatment of patients with HCC who have been previously treated with sorafenib. As with nivolumab monotherapy, approval was based on the results of the single‐arm phase 1/2 CheckMate 040 trial. With the ipilimumab plus nivolumab dosing regimen subsequently approved by the FDA, the overall ORR was 32% and the median duration of response was 17.5 months. The combination was also found to be generally well tolerated.^71^ A phase 3 trial of ipilimumab plus nivolumab vs sorafenib or lenvatinib in the first‐line setting is currently recruiting participants (CheckMate 9DW; NCT04039607).

Pembrolizumab is, like nivolumab, a recombinant anti‐PD‐1 monoclonal antibody that is administered by intravenous infusion. Pembrolizumab was approved by the FDA for the treatment of patients with HCC who have been previously treated with sorafenib on the basis of the results of the single‐arm phase 2 KEYNOTE‐224 trial.^31^, ^72^ In KEYNOTE‐224, the ORR and DCR with pembrolizumab were 17% and 62% respectively. However, despite directionally favourable results (improved median OS and PFS), the randomized, parallel‐assignment, double‐blind phase 3 KEYNOTE‐240 trial of pembrolizumab vs placebo in participants with advanced HCC who were previously treated with sorafenib did not meet statistical significance as defined in the pre‐specified statistical plan in either of its co‐primary endpoints of OS (HR 0.78, 95% CI 0.611‐0.998; *P*=0.0238) or PFS (HR 0.78, 95% CI 0.61‐0.99; *P*=0.0186).^20^

After the negative results from two randomized controlled trials with PD‐1 monotherapy in the first‐ and second‐line setting, and in anticipation of the approval of the combination of atezolizumab plus bevacizumab in the first‐line setting, it is unclear how the FDA will deal with the approval of nivolumab and pembrolizumab in the second‐line setting. The results of a phase 2 trial, in Chinese patients with advanced HCC who had progressed on or were intolerant to previous systemic treatment, suggest camrelizumab, another anti‐PD1 antibody, might represent an additional treatment option.^73^

## 3 FACTORS INFORMING TREATMENT DECISIONS

Approximately one‐quarter to one‐third of patients with advanced HCC are eligible for second‐line systemic treatment, based on their liver function and comorbidities. An analysis of real‐world data from patients in Canada with HCC found that 13.1% and 31.7% were eligible, based on strict and modified eligibility criteria respectively, for regorafenib, cabozantinib or ramucirumab after sorafenib.^74^ Patients with advanced HCC and preserved liver function can now survive with sequential systemic treatment for more than 2 years;^34^ key to this outcome is optimizing the patient journey, including the timing and sequencing of treatment switches. Additional guidance on when to switch from first‐line to second‐line treatment would likely be useful for many clinicians. Decisions will be influenced by whether discontinuation of first‐line treatment is due to AEs or loss of clinical benefit and whether the disease has progressed, either radiologically or symptomatically, and, if radiologically, the pattern of progression.^75^ In contrast to current clinical trials, which typically require only radiological progression under systemic first‐line therapy to allow switch to another therapy, in clinical practice circumscribed intrahepatic progression (small increases in the diameters of existing intrahepatic lesions or a new small hepatic lesion) will not, in some cases, be a definite reason to switch. Liver function is an important consideration, meaning that patients should also not be treated too long with transarterial approaches before switching,^76^ and patients who are not eligible for transarterial chemoembolization should be identified as early as possible. Conversely, physicians should not switch treatment too soon (e.g. before disease progression or in the absence of intolerable treatment‐related AEs) or sequential treatment benefit may be lost.

In choosing from among the available second‐line systemic treatment options, clinicians will consider efficacy, safety and tolerability, HRQoL, route of administration, dosing regimen, drug class, molecular targets and individual patient’s characteristics (see Tables 1, 2, 3 for summaries). An additional consideration may be that all second‐line treatment options are approved for patients previously treated with sorafenib and whether they are therefore suitable for use to treat patients previously treated with another first‐line systemic treatment, such as lenvatinib. In an exploratory *post hoc* analysis of data from the REFLECT trial, median OS was 25.7 months in patients who responded to lenvatinib and received any subsequent anticancer medication, 26.2 months in patients who responded to lenvatinib and subsequently received sorafenib, and 22.3 months in patients who responded to sorafenib and received any subsequent anticancer medication.^34^

Given the differences between study populations in the pivotal clinical trials, clinicians can, to some extent, align treatment decisions with key patient characteristics. For example, ramucirumab is not indicated for patients with AFP <400 ng/mL, and regorafenib may not be suitable for patients who were sorafenib‐intolerant (although this can be difficult to define in routine clinical practice), whereas cabozantinib may be appropriate as an all‐comer drug for both of these groups and as both a second‐ and third‐line treatment option (Table 4). Treatment with ipilimumab plus nivolumab was associated with a high ORR in CheckMate 40, suggesting it may emerge as a preferred treatment option for a subgroup of patients with high‐burden progression. The choice of second‐line treatment is independent of the aetiology of the underlying liver disease or tumour stage and the response to previous sorafenib treatment. Furthermore, the presence of comorbidities may add weight to consideration of AE profiles. It should therefore be noted, for example, that the USA prescribing information for regorafenib contains a black box warning for severe hepatotoxicity.^24^ Route and schedule of administration are also important for many patients and may impact HRQoL and treatment adherence.

**Table 4:** Some considerations when choosing second‐ or third‐line systemic treatment for patients with advanced HCC in clinical practice

	Second‐line treatment		Third‐line treatment
	Sorafenib‐intolerant patients	Patients with low AFP	
Regorafenib	No	Yes	No
Cabozantinib	Yes	Yes	Yes
Ramuciramab	Yes	No	No

Abbreviations: AFP, alpha‐fetoprotein; HCC, hepatocellular carcinoma.

## 4 OPEN ISSUES

### 4.1 Biomarkers

Identification of biomarkers is crucial in defining, stratifying and selecting subgroups of patients who can benefit most from different treatments. Therefore, collection of tumour samples and liquid biopsy should be mandatory to identify both prognostic and predictive biomarkers that will help clinicians to better tailor treatment for patients with advanced HCC. At present, biomarker‐driven patient selection is possible only for ramucirumab. Potential prognostic biomarkers include MET for cabozantinib,^45^ among many others, but no predictive biomarkers have yet been identified. An evaluation of outcomes in CELESTIAL based on 13 biomarkers, with baseline levels dichotomized at the median, found that cabozantinib treatment was associated with improved OS and PFS vs placebo in previously treated advanced HCC irrespective of baseline biomarker levels; low baseline levels of MET, hepatocyte growth factor, growth arrest‐specific protein 6, VEGF‐A, angiopoietin‐2 and interleukin‐8 and high levels of insulin‐like growth factor 1 were identified as potential prognostic biomarkers for longer OS with placebo.^77^ Plasma proteins and microRNAs are potential prognostic biomarkers for regorafenib (some may be predictive, but none can be used as such in clinical practice currently).^78^

Programmed death‐ligand 1 (PD‐L1) expression levels were assessed retrospectively as a potential biomarker for nivolumab therapy in CheckMate 040, using a cut‐off of membrane expression of PD‐L1 on ≥1% of tumour cells. No association with objective responses was found but the authors noted that in‐depth characterization of tumour‐infiltrating T‐cell and macrophage subsets, including their expression of PD‐1 and PD‐L1, could be valuable for biomarker assessments in patients with advanced HCC.^69^ In KEYNOTE‐224, higher PD‐L1 immunohistochemistry combined positive scores (a measure of PD‐L1‐positive immune and tumour cells) was associated with better response to treatment with pembrolizumab,^79^ although this association has not yet been further confirmed.

### 4.2 Combination therapies under investigation (e.g. antiangiogenics/tyrosine kinase inhibitors plus immunotherapy)

There are strong rationales for combining ground‐breaking immunotherapeutic agents with tyrosine kinase inhibitors or VEGF inhibitors. For example, it has been hypothesized that alleviating tumour hypoxia could be a valuable approach to improving the outcomes associated with current immunotherapies. Therefore, concurrent targeting of VEGF and its cognate receptors and immune checkpoints may be effective, and this hypothesis is now supported by pre‐clinical and clinical data. For example, and as described above, atezolizumab (an immunotherapeutic agent targeting PD‐L1) in combination with bevacizumab (an inhibitor of VEGF‐A) has been found to offer improvements in OS and PFS vs sorafenib, in patients with unresectable HCC, who had not received prior systemic therapy.^14^ It seems likely that atezolizumab plus bevacizumab will become the standard treatment in the first‐line setting for patients with advanced HCC in the near future, fundamentally changing the current paradigm. It might be speculated that following resistance or intolerance to, or progression on atezolizumab plus bevacizumab, many clinicians will often use a current first‐line treatment option, such as sorafenib or (off‐label) lenvatinib, in the second‐line setting. Cabozantinib may continue to be used in the second‐line setting (off‐label without prior sorafenib treatment) but that seems less likely for regorafenib, owing to the lack of data from patients who did not tolerate sorafenib, and ramucirumab, owing to similarities to bevacizumab (both being monoclonal antibodies that target VEGFs).

Also under investigation as first‐line treatments for patients with advanced HCC in phase 3 trials are the combinations of lenvatinib plus pembrolizumab, vs lenvatinib,^80^ of cabozantinib plus atezolizumab, vs sorafenib, vs cabozantinib,^81^ of durvalumab plus tremelimumab (two checkpoint inhibitors), vs durvalumab, vs sorafenib,^82^ and of nivolumab and ipilimumab vs sorafenib or lenvatinib (NCT04039607). Combination therapies are likely to further increase the complexity of the treatment landscape for patients with advanced HCC, especially regarding sequential treatment approaches.

## 5 CONCLUSION

There is limited evidence to support clinicians in choosing between approved second‐line treatments for patients with advanced HCC, particularly in terms of sequencing. While product characteristics, and trial data on the efficacy, safety and tolerability and HRQoL outcomes associated with different treatment options can and should be used to inform the decision, until more data become available on current and new treatment pathways the single most important factor remains the individual patient, and their preferences should be closely considered.

## Conflict of interest

Lorenza Rimassa has worked in a consulting or advisory role with Amgen, ArQule, Baxter, Basilea, Bayer, Celgene, Eisai, Exelixis, Hengrui Therapeutics, Incyte, Ipsen, Italfarmaco, Lilly, Merck Sharp & Dohme, Roche, Sanofi and Sirtex Medical, has received lecture fees from AbbVie, Amgen, AstraZeneca, Eisai, Gilead, Ipsen, Lilly, Roche and Sanofi, travel expenses from ArQule and Ipsen, and institutional research funding from Agios, ARMO BioSciences, AstraZeneca, BeiGene, Eisai, Exelixis, Incyte, Ipsen, Lilly and Merck Sharp & Dohme. Marcus‐Alexander Wörns has received consultancy honoraria from AbbVie, Bayer, Bristol‐Myers Squibb, Eisai, Ipsen and Roche, lecture fees from AbbVie, Bayer, Bristol‐Myers Squibb, Celgene, Gilead Sciences, Incyte, Ipsen, Janssen‐Cilag and Merck Sharp & Dohme, and conference fees/travel expense reimbursement from AbbVie, Bayer, Bristol‐Myers Squibb, Gilead Sciences and Ipsen.

## Supporting information

Table S1‐S2
