# Supplementary material for: Navigating the new landscape of second‐line treatment in advanced hepatocellular carcinoma
Source: Liver Int. 2020 Jun 10;40(8):1800–11. doi: 10.1111/liv.14533 (PMC7496898; doi:10.1111/liv.14533)
Supplement: Supplementary file 1 — Table S1‐S2 [file LIV-40-1800-s001.docx]

**SUPPLEMENTAL TABLE 1** Overview of second-line phase 3 trials with a negative outcome

| **Treatment comparison (study)** | **Target(s)** | **Patients (N)** | **OS results** |
| --- | --- | --- | --- |
| Brivanib vs placebo (BRISK-PS)^15^ | VEGFR, FGFR | Previous sorafenib (395) | 9.4 vs 8.2 months HR 0.89 (95% CI 0.69–1.15), p = 0.33 |
| Everolimus vs placebo  (EVOLVE-1)^19^ | mTOR | Previous sorafenib (546) | 7.6 vs 7.3 months  HR 1.05 (95% CI 0.86–1.27), p = 0.68 |
| Ramucirumab vs placebo  (REACH)^23^ | VEGFR2 | Intolerant of or failed sorafenib (565) | 9.2 vs 7.6 months  HR 0.87 (95% CI 0.72–1.05), p = 0.14 |
| ADI-PEG 20 plus BSC vs placebo^16^ | ADI | Prior systemic therapy (635) | 7.8 vs 7.4 months  HR 1.02 (95% CI 0.847–1.233), p = 0.88 |
| Tivantinib vs placebo (METIV-HCC)^17^ | MET | Previous sorafenib and high MET (340) | 8.4 vs 9.1 months  HR 0.97 (95% CI 0.75–1.25), p = 0.81 |
| Tivantinib vs placebo (JET-HCC)^18^ | MET | Previous sorafenib and high MET (386) | 9.9 vs 8.5 months  HR 0.85 (95% CI 0.59–1.22), p = not given |
| Pembrolizumab plus BSC vs placebo plus BSC (KEYNOTE-240)^20^ | PD-1 | Previous sorafenib (413) | HR 0.78 (one sided p = 0.0238; not significant as per the prespecified statistical plan) |
| S-1 (chemotherapeutic agent based on fluorouracil) vs placebo^21^ | Chemotherapeutic agent | Previous sorafenib (334) | 11.1 vs 11.2 months  HR 0.86 (95% CI 0.67–1.10), p = 0.220 |
| Doxorubicin-loaded nanoparticles vs SoC^22^ | Chemotherapeutic agent | Previous sorafenib (397) | 9.1 vs 9.0 months  HR 1.00 (95% CI 0.78–1.28), p = 0.99 |

ADI, arginine deaminase; BSC, best supportive care; CI, confidence interval; FGFR, fibroblast growth factor receptor; HCC, hepatocellular carcinoma; HR, hazard ratio; mTOR, mammalian target of rapamycin; PD-1, programmed death receptor-1; SoC, standard of care; VEGFR, vascular endothelial growth factor receptor
